# Workplace stressors as a mediating mechanism between social support and depression among Myanmar migrant workers in Thailand: A structural equation modeling approach

**DOI:** 10.1016/j.jmh.2026.100400

**Published:** 2026-01-28

**Authors:** Nanda Win, Nuchanad Hounnaklang, Pankaew Tantirattanakulchai

**Affiliations:** a Faculty of Public Health, Thammasat University, Pathum Thani 12121, Thailand; b College of Public Health Sciences, Chulalongkorn University, 11 floor, Sabbasastravicaya Building, Soi Chulalongkorn 62, Phyathai Road, Pathumwan, Bangkok, 10330, Thailand; c Department of Health Education and Behavioral Sciences, Faculty of Public Health, Mahidol University, 420/1, Ratchawithi Road, Ratchathewi, Bangkok, 10400, Thailand

**Keywords:** Depression, Social support, Workplace stressors, Migrant workers

## Abstract

**Introduction:**

Depression is a major global public health challenge and a leading cause of disability worldwide, with a particularly high and concerning burden among migrant workers. This research objective is to discover the association between social support and depression, and the mediating role of workplace stressors.

**Methods:**

A cross-sectional survey was conducted via simple random sampling among 500 Myanmar migrant workers in nine factories. Workplace stressor, The Interpersonal Support Evaluation List-12, and The Center for Epidemiological Studies-Depression were used to collect the data. Mplus version 8.7 was used to construct the structural equation model.

**Results:**

Among 500 total participants, the prevalence of depression was around 47%. The SEM good fit with the data (χ2/df = 3.526, RMSEA = 0.071, CFI = 0.972). Social support had negatively direct effect on workplace stressor (*β* = 0.175, *p* < 0.001) as well as on depression (*β* = 0.078, *p* < 0.05). Workplace stressor had positively direct effect on depression (*β* = 0.787, *p* < 0.001). Social support negatively indirect effect on depression mediating through workplace stressor (*β* = 0.138, *p* < 0.001).

**Conclusion:**

These findings indicated that workplace stressors and social support are important variables that effect on depression among Myanmar migrant workers in Thailand. Therefore, health policymakers need to adopt some preventive measures for improving social support and creating a healthy working environment for migrant workers.

## Highlights

• Depression is highly prevalent among migrant workers globally.
• Greater social support reduces depression among migrant workers.
• Workplace stressors play a mediating role between social support and depression.
• Public policies should enhance support and promote healthy workplaces to reduce depression.

## 1 Introduction

In the past few decades, it has risen to 281 million migrants around the world, which was 3.6 % of the world's population. Human migrating from one place to another has impacted on both developed and developing nations (International Organization for Migration, 2021). In 2006, the total number of migrant workers was around 200 million and is predicted to 230 million until 2050 globally (United Nations, 2004) In Thailand, over 3.9 million migrants is constituting about 10 % of the labor force (Khai, 2023). The majority of these migrants are from neighboring countries including Cambodia, Laos, and Myanmar who are under skill level hard labor workers. Additionally, it has been steadily increasing, with 5 million. At least 1.4 million are Myanmar migrant workers, in which just 350,000 of them are in employment through the official Memorandum of Understanding between Myanmar and Thailand and others were unregistered migrant workers (International Labour Organization, 2023). The influx of migrants began in the 1990s to meet the demand for labor due to rapid economic growth (Guinto et al., 2015). These migrants face various challenges, including limited access to healthcare and social protection, highlighting the need for improved policies and services to cater to their needs. These can have an impact on health physically, mentally, and socially. Therefore, it leads to depression (Gkiouleka et al., 2018). According to one study among 445 of Myanmar migrants in Thailand, the prevalence of depression was around 11.9 % of the participants reported symptoms of depression (Kesornsri et al., 2019).

Globally, depression affects approximately 121 million individuals and is recognized as one of the most prevalent psychological disorders. According to the World Health Organization (WHO), it is the leading cause of ill health and disability worldwide and ranks as the fourth leading contributor to the global burden of disease (Huijts et al., 2017; World Health Organization, 2017) Currently, it is the second leading cause of disability-adjusted life years (DALYs) among individuals aged 15 to 44 years (Reddy, 2010). It is widely known as the primary cause of suicides and suicide attempts. Certainly, 40 % to 80 % of all suicide attempts are due to depression worldwide (Essayagh et al., 2023). One of the main causes for having depression among migrant worker is workplace stressor. In the working environment, the psychological health of workers is a main issue, because it can reduce work production and affect their stress levels. As a result, these stressed hard labor workers have higher chance to experience work disruptions and accidents (Kim et al., 2017; Park et al., 2004). Work-related stress has been investigated correlated with depressive symptoms in migrant workers (Chen et al., 2009). Throughout the process of working, migrant workers having experienced discrimination, long working durations, and poor working environments and low salaries leading to get more depression.

An important protection for depression is social support. Social support plays a crucial role in reducing the risk of depression. Depression in migrant populations is influenced by social support. From stress-buffering model, social support may decrease adverse psychological impacts on mental health (Schwarzer and Knoll, 2007). Multiple studies have shown that higher levels of perceived social support are associated with lower incidence of depressive symptoms (Choi et al., 2023). According to one of the South Korea study, high social support can decrease depression level (Jung and Kim, 2020). Due to the direct effect of social support on mental health, perceived social support significantly did the moderating effect of job stressors on depression (Chen et al., 2009). Different types of social support, such as emotional/informational support, positive social interactions, and tangible support, have been found to be particularly beneficial in protecting against depression (Silva et al., 2023). Specifically, perceived social support from family, friends, and significant others has been found to be important in reducing depression among migrants (Li et al., 2022; Kim et al., 2022; Postali, 2022).

Therefore, the objective of the study was to investigate the effect of social support on depression among Myanmar migrant workers and further explore the mediating role of workplace stressor for providing some theoretical guidance for future studies and interventions of depression in Myanmar migrant workers.

## 2 Material and methods

### 2.1 Study design and participants

A study design was a cross-sectional study design conducting on 500 Myanmar migrant factory workers in nine industries in Samut Prakan Province, Thailand. A multi-stage sampling approach was used to select the study sites. First, the Mueang Samut Prakan district was purposively selected due to its high density of industrial sites (2043 factories) and large population of Myanmar migrant workers. Second, the factories within this district were stratified by size (small, medium, and large) based on investment capital and workforce size. Finally, simple random sampling was used to select nine factories from the small and medium-sized strata. From these selected factories, a total pool of 500 Myanmar migrant workers was identified. Self-administered method of data collection was done. Myanmar migrant factory workers between 18–50 years old, having legalized in working in Thailand, and living in Thailand >6 months were selected to include in the study. The exclusion criteria are Myanmar migrant factory workers with serious physical and mental impairment and were not willing to join the study.

### 2.2 Measurements

#### 2.2.1 Socio-demographic characteristics

The sociodemographic variables included age, gender, marital status and educational level.

#### 2.2.2 Workplace stressors

Workplace stressor questionnaire consists of 18 items for measuring coercive working conditions, daily hassles, and barriers to resign job (Meyer et al., 2016). It was a 5 Likert scale from 0 (Never) to 4 (Always). The score ranges from 0 to 72. Cronbach’s alpha was 0.82.

#### 2.2.3 Social support

The Interpersonal Support Evaluation List-12 (ISEL-12) was used to measure social support (Kesornsri et al., 2019). The scale is composed of 12 items. It measures three dimensions of support including appraisal support, belonging support and tangible support from family, friends and significant others. It uses 4 Likert Scale including (1) definitely false, (2) probably false, (3) probably true, (4) definitely true. The total score was 0–36. Cronbach’s alpha was 0.83.

#### 2.2.4 Depression

The Center for Epidemiological Studies-Depression (CES-D) was used to measure depression. The number of questions was 20 items with 5-point Likert scale from 0 (rarely or none of the time) to 3 (most or almost all the time). Four dimensions including (1) depressed affect, (2) somatic complaints, (3) positive affect, and (4) interpersonal activity were measured (Radloff, 1977). The score ranges from 0 to 60. Cut-off point was 16. Cronbach’s alpha was 0.89.

### 2.3 Ethics approval and informed consent

This study was approved by The Research Ethics Review Committee for Research Involving Human Research Participants, Group I, Chulalongkorn University (COA No. 143/66) on July 1, 2023. Written consent was obtained to protect the identities of the participants and maintain confidentiality before the data collection.

### 2.4 Statistical analysis

SPSS version 29.0 was used for analyzing the descriptive statistical analysis and correlation analysis. To confirm the mediating effect of workplace stressor on the relationship between social support and depression, the Structural Equation Model (SEM) was applied by using Mplus 8.8.

The model fitted with the SEM criteria: χ^2^/df < 4 (Kline, 2005), Comparative Fit Index (CFI) > 0.900 (Hu and Bentler, 1999), Tucker-Lewis Index (TLI) > 0.900 (Hu and Bentler, 1999), Root Mean Square Error of Approximation (RMSEA) < 0.080 (Schreiber et al., 2006), and Standardized Root Mean Squared Residual (SRMR) < 0.080 (Schreiber et al., 2006). For determining the best fit of model, the smallest Akaike information criterion (AIC) and Bayesian information criterion (BIC) were used (Schermelleh-Engel et al., 2003).

## 3 Results

The socio-demographic characteristics of Myanmar migrant factory workers indicated that the mean age of participants was 28 ± 7 years. The sample comprised a greater proportion of men than women, and 70.6 % were single. In terms of education, 48.0 % had completed primary school. Regarding mental health, 47.0 % of participants reported experiencing symptoms of depression (Table 1).

**Table 1:** Characteristics of Myanmar migrant workers (*n* = 500).

Variables	Number	Percentage
**Age (years)**		
18–27	237	47.4
28–37	195	40.4
38–45	61	12.2
Range	18–45	
Mean (SD)	28.7 (7.1)	
**Sex**		
Female	228	45.6
Male	272	54.4
**Marital status**		
Single	382	70.6
Married	115	23.0
Divorced/Separated	3	6.4
**Educational level**		
No education	29	5.8
Primary school	240	48.0
Middle/High school	231	46.2

Note**:** SD, Standard deviation

The correlation analysis demonstrated strong positive associations among the three types of social support: appraisal, belonging, and tangible support (*r* = 0.815 to 0.860, *p* < 0.01), indicating good internal consistency. Social support was negatively correlated with workplace stressors and psychological distress, including workplace stress (*r* = –0.141), coercive working conditions (*r* = –0.115), daily hassles (*r* = –0.157), and depression (*r* = –0.178), all significant at *p* < 0.01. Coercive working conditions were highly correlated with daily hassles (*r* = 0.894) and barriers to resigning (*r* = 0.944). Depression showed strong positive correlations with negative affect (*r* = 0.869), somatic symptoms (*r* = 0.720), and interpersonal difficulties (*r* = 0.608), and a moderate negative correlation with positive affect (*r* = –0.470), supporting its multidimensional structure (Table 2).

**Table 2:** Inter-correlation matrix of study variables.

	SS	AS	BS	TS	WS	CW	DH	BR	DP	PA	NA	SR	ID
SS	1.000												
AS	0.848⁎⁎	1.000											
BS	0.860⁎⁎	0.628⁎⁎	1.000										
TS	0.815⁎⁎	0.516⁎⁎	0.537⁎⁎	1.000									
WS	−0.141⁎⁎	−0.078	−0.183⁎⁎	−0.094*	1.000								
CW	−0.115⁎⁎	−0.053	−0.154⁎⁎	−0.080	0.894⁎⁎	1.000							
DH	−0.157⁎⁎	−0.093*	−0.186⁎⁎	−0.117⁎⁎	0.976⁎⁎	0.806⁎⁎	1.000						
BR	−0.104*	−0.055	−0.164⁎⁎	−0.042	0.944⁎⁎	0.799⁎⁎	0.886⁎⁎	1.000					
DP	−0.178⁎⁎	−0.127⁎⁎	−0.222⁎⁎	−0.098*	0.711⁎⁎	0.553⁎⁎	0.731⁎⁎	0.673⁎⁎	1.000				
PA	−0.143⁎⁎	−0.105*	−0.128⁎⁎	−0.127⁎⁎	0.274⁎⁎	0.210⁎⁎	0.272⁎⁎	0.280⁎⁎	0.470⁎⁎	1.000			
NA	−0.141⁎⁎	−0.111*	−0.189⁎⁎	−0.057	0.635⁎⁎	0.496⁎⁎	0.655⁎⁎	0.593⁎⁎	0.869⁎⁎	0.161⁎⁎	1.000		
SR	−0.066	−0.023	−0.146⁎⁎	0.003	0.561⁎⁎	0.446⁎⁎	0.574⁎⁎	0.525⁎⁎	0.720⁎⁎	−0.120⁎⁎	0.631⁎⁎	1.000	
ID	−0.138⁎⁎	−0.123⁎⁎	−0.122⁎⁎	−0.103*	0.430⁎⁎	0.309⁎⁎	0.463⁎⁎	0.385⁎⁎	0.608⁎⁎	0.019	0.563⁎⁎	0.419⁎⁎	1.000
**M**	25.12	8.26	8.37	8.49	8.66	2.55	4.05	2.06	14.50	4.48	3.62	5.13	1.27
**SD**	6.75	2.63	2.70	2.69	4.68	1.12	2.53	1.28	7.74	3.49	3.27	3.34	1.40

**Notes:** M, mean; SD, standard deviation; SS, social support; AS, appraisal support; BS, belonging support; TS, tangible support, WS, workplace stressor; CE, coercive working conditions; DH, daily hassles; BR, barrier to resign job; DP, depression; PA, positive affect; NA, negative affect; SR, somatic symptoms and retarded activity; ID, interpersonal difficulties. *p < 0.05.

⁎⁎ *p* < 0.01.

The structural equation model demonstrated a good overall fit to the data, as reflected by the fit indices: χ²/df = 3.526, RMSEA = 0.071, SRMR = 0.041, CFI = 0.972, and TLI = 0.958. All values satisfied the commonly accepted thresholds, indicating that the hypothesized model adequately represented the observed data (Table 3).

**Table 3:** The fitness indicators of structural equation model.

Fitness Indicators	χ^2^ /df	RMSEA	SRMR	CFI	TLI	AIC	BIC
Reference value	≤4	<0.080	<0.080	>0.900	>0.900	-	-
Correction value	3.526	0.071	0.041	0.972	0.958	19801.459	19948.970

**Notes:** χ^2^, chi-square; df, degrees of freedom; RMSEA, root mean square error of approximation; SRMR, standardized root mean squared residual; CFI, comparative fit index; TLI, Tucker-Lewis index; AIC, Akaike information criterion; BIC, Bayesian information criterion.

**Fig. 1:**
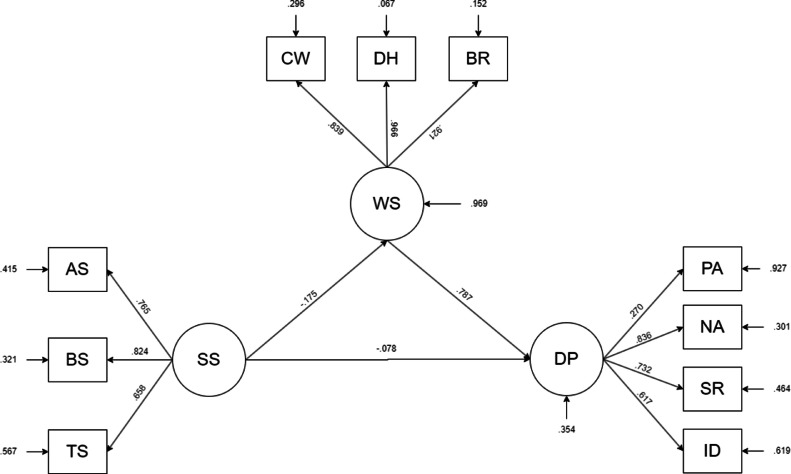
Structural equation model for depression of Myanmar migrant workers in Thailand.

Notes: SS, social support; AS, Appraisal support; BS, Belonging support; TS, Tangible support; WS, workplace stressor; CW, Coercive working conditions; DH, Daily hassles; BR, Barrier to resign job; DP, depression, PA, Positive affect; NA, Negative affect; SR, Somatic symptoms and retarded activity; ID, Interpersonal difficulties.

Path analysis revealed both direct and indirect relationships among the study variables. Social support demonstrated significant negative associations with workplace stressors (β = –0.175, *p* < 0.001) and depression (β = –0.078, *p* = 0.042). Workplace stressors exhibited a strong positive association with depression (β = 0.787, *p* < 0.001) (Table 4). Additionally, social support had a significant indirect negative effect on depression via workplace stressors (β = –0.138, *p* < 0.001), supporting a partial mediation effect. All structural pathways are depicted in Fig. 1.

**Table 4:** Test results of path relationship.

Model Pathway	Standardized Estimate	S.E.	Est / S.E.	95 % CI		*P*
				**Lower**	**Upper**	
**Direct effects model**						
WS ← SS	−0.175	0.050	−3.512	−0.257	−0.093	<0.001
DP ← SS	−0.078	0.038	−2.030	−0.141	−0.015	0.042
DP ← WS	0.787	0.024	33.142	0.748	0.826	<0.001
**Indirect effects model**						
DP← WS ← SS	−0.138	0.039	−3.513	−0.202	−0.073	<0.001

**Notes:** Est, Estimate**;** S.E., Standard Error; CI, Confidence Interval; *P*, P-value; SS, social support; WS, workplace stressor; DP, depression.

## 4 Discussion

The results from our study discovered that 47 % of participants had depression. The result was higher than the findings reported from Aung et al. (13 %) (Aung et al., 2019), and Kesornsri et al. (11.9 %) (Kesornsri et al., 2019). The inconsistency in the depression rate could be due to using of different measurements or samples. Another reason may be due to after COVID-19 situations. The post-acute sequelae of COVID-19 (PASC) shows the long-term symptoms which remain after having COVID-19. Depression is one of the common PASC symptoms (Taquet et al., 2021). In addition, these workers face health risks, occupational hazards, and challenges related to training and management. Issues like inadequate training, health problems, and labor relations impact their work environment and effect on their mental health (Wannapa Luekitinan, 2015; Auethavornpipat, 2022).

First, according to the findings that were reported herein, social support could directly predict on having depression or not to Myanmar migrant factory workers. Social support plays a crucial role in reducing depression. Research indicates that social support could prevent stressors, reducing the likelihood of clinical depression and other forms of psychopathology (Lin and Dean, 1984; Brown and Andrews, 1986). A meta-analysis of 64 researches on the association between social support and depression provided a strong relationship between social support and depression among workers (Harandi et al., 2017). Therefore, having higher social support from family, friends, other significant others could increase the protective factor for mental illness among migrant workers and improve their psychological resilience. On the other hand, low social support and other factors such as discrimination and far away from family members may cause depression among migrants (Björkman et al., 2011; Jurado et al., 2017).

The second conclusion was that social support had a direct effect on workplace stressors. Workplace stressors, such as job-related stress, can significantly impact employee performance (Tehreem et al., 2023). Studies have shown that social support, both from work and non-work-related sources, can act as a buffer against job stress and work-family conflict, reducing their adverse impacts. Encouraging coworker support has been linked to a reduction in psychological distress among employees, highlighting the significance of fostering supportive relationships at workplace for mental health and well-being.

Thirdly, workplace stressors significantly impact the mental health of migrant workers, causing depression (Umar et al., 2023; Li et al., 2019b; Cao et al., 2022; Li et al., 2019a; Poudel et al., 2019). Throughout the process of working, migrant workers having experienced discrimination, long working durations, and poor working environments and low salaries leading to get more depression (Robert et al., 2014). In summary, this study pointed out that workplace stressor was a mediating factor between social support and depression. The result was inconsistent with one previous study in which it was unsignificant about social support being a mediating factor between workplace stressor and depression (Kim et al., 2022). From one cross-sectional study among 843 participants in China, social support was negatively related to depression, and moderated the negative impact of job stressors on depression (Chen et al., 2009). According to our study result, it means that depression was further decreased by getting higher social support from family, or friends during working in low stress workplace situation. In other words, it was demonstrated that the impact of social support on depression was greater in the group having low workplace stressor than in the group having high workplace stressor.

Although Social Support showed a statistically significant association with depression, the magnitude of this relationship was relatively weak within the model. From a theoretical perspective, this pattern is consistent with the stress-buffering hypothesis, which suggested that social support primarily influenced mental health indirectly by mitigating the impact of stress rather than exerting a strong direct effect (Acoba, 2024). In line with this framework, the present findings indicated that social support operated mainly through the reduction of workplace stressors, which emerged as the dominant pathway to depression.

From a contextual standpoint, migrant workers are often exposed to persistent structural and occupational stressors, such as job insecurity, long working hours, and limited labor protections. Under such conditions, social support alone may be insufficient to directly offset depressive symptoms, thereby resulting in a comparatively weaker direct association with mental health outcomes (Sumerlin et al., 2025). Methodologically, the modest effect size of social support may also reflect its multidimensional nature, whereby different sources of support vary in their relevance and effectiveness in influencing depression. Together, these considerations suggest that the weak but significant association of social support reflects its role as a secondary essential protective factor operating within a broader stress-driven process.

### 4.1 Limitations

This research has some limitations. First, the study design was a cross-sectional study, and any causal relationship should be inferred cautiously based on the association observed in our study. Thus, future studies should consider a longitudinal study design to further understand the causal and temporal associations between these variables. Secondly, depression is the complex construct in which a good number of potentially confounding data and variables have not been captured in this study. Third, regarding generalizability, this study was conducted only in Samut Prakan Province, which may not represent migrant workers in other regions of Thailand. Furthermore, because we purposively selected the Mueang Samut Prakan district and focused on small-to-medium sized factories, the results may not fully represent the diverse experiences of workers across all factory types or locations within the province itself. Although we used random sampling to select factories within the chosen district, the specific characteristics of this industrial hub may differ from other areas. Additionally, this study gathered data solely through self-report questionnaires, which may influence the accuracy of the findings, as some participants may not answer the sensitive questions truthfully. It is possible to enhance the reliability of the findings obtained from future research by utilizing a methodology that combines self-reports with objective indicators.

## 5 Conclusions

Overall, this study improves understanding of factors related to depression among migrant workers by examining workplace stressors and social support. The results show that workplace stressors have a much stronger effect on depression and represent the main pathway linking psychosocial conditions to mental health outcomes. In contrast, social support has a smaller but statistically significant effect and mainly helps reduce stress. These findings indicate that social support acts as a secondary protective factor rather than a strong direct determinant of depression. Therefore, interventions should focus primarily on reducing workplace stressors, while also strengthening social support to improve migrant workers’ mental health.

## Funding source

This research did not receive any specific grant from funding agencies in the public, commercial, or not-for-profit sectors.

## CRediT authorship contribution statement

**Nanda Win:** Conceptualization, Data curation, Formal analysis, Investigation, Methodology, Project administration, Validation, Visualization, Writing – original draft, Writing – review & editing. **Nuchanad Hounnaklang:** Conceptualization, Formal analysis, Methodology, Supervision, Validation, Writing – original draft, Writing – review & editing. **Pankaew Tantirattanakulchai:** Conceptualization, Data curation, Formal analysis, Investigation, Methodology, Project administration, Software, Writing – original draft, Writing – review & editing.

## Declaration of competing interest

The authors declare that they have no known competing financial interests or personal relationships that could have appeared to influence the work reported in this paper.
